# Supplementary material for: OPA1 controls mitochondrial dysfunction-driven liver fibrosis in MASLD
Source: bioRxiv. 2026 Sep 27:2026.09.24.753666. Preprint. [Version 1] doi: 10.64898/2026.09.24.753666 (PMC13622249; doi:10.64898/2026.09.24.753666)
Supplement: Supplementary file 1 [file media-1.pdf]

Supplementary materials for manuscript:

"OPA1 controls mitochondrial dysfunction-driven liver fibrosis in MASLD" by Xinting Yu, Pinzhu Huang, Arvo Justice, Aaron Hakim, Yoshiko Nanki, Zaur Abilov, Edward O'Neill, Jerry Cruz Rodriguez, Enzo Cabrera, Marla Sagatelian, Chandra S Shankar, Brenda Flam, Kawtar Al Khalloufi, Hiromi Sesaki, Shisong Rong[#], Yury V. Popov[#]

# Correspondence to:

Yury Popov MD, PhD
Division of Gastroenterology, Hepatology and Nutrition
Beth Israel Deaconess Medical Center, Harvard Medical School
99 Brookline Ave., RN-234
ypopov@bidmc.harvard.edu, yury.popov@gmail.com

or:

Shisong Rong MD, PhD
Department of Ophthalmology, Ocular Genomics Institute, Mass Eye and Ear, Mass General Brigham, Harvard Medical School, Boston, MA, United States
243 Charles St., Boston, 02114 MA, USA
Shisong_rong@meei.harvard.edu, Shisong_rong@outlook.com

Table S1. Genome-wide association studies included in the Multi-Trait Analysis of GWAS

	Author (Year)	Traits	Ancestry	Sample size	Mean χ²	λGC	LDSC Intercept (SE)	Attenuation Ratio	Ref.
1	Sun Z (2023)	MASLD	European	32,941	1.0574	1.0466	0.9976	-0.042	[1]
2	Ghodsian N (2021)	MASLD	European	778,614	1.0311	1.0285	1.0080	0.257	[2]
3	Fairfield CJ (2021)	MASLD	European	413,256	1.0781	1.0679	1.0089	0.114	[3]
4	Kurki MI (2023)	MASLD	European (Finnish)	500,348	1.0749	1.0527	1.0194	0.259	[4]
5	Ghouse J (2024)	Cirrhosis of liver	Mixed ethnicity	3,179,403	1.1449	1.1082	1.0226	0.156	[5]
6	Ghouse J (2024)	Cirrhosis of liver	European	2,039,708	1.1486	1.1144	1.0268	0.180	[5]

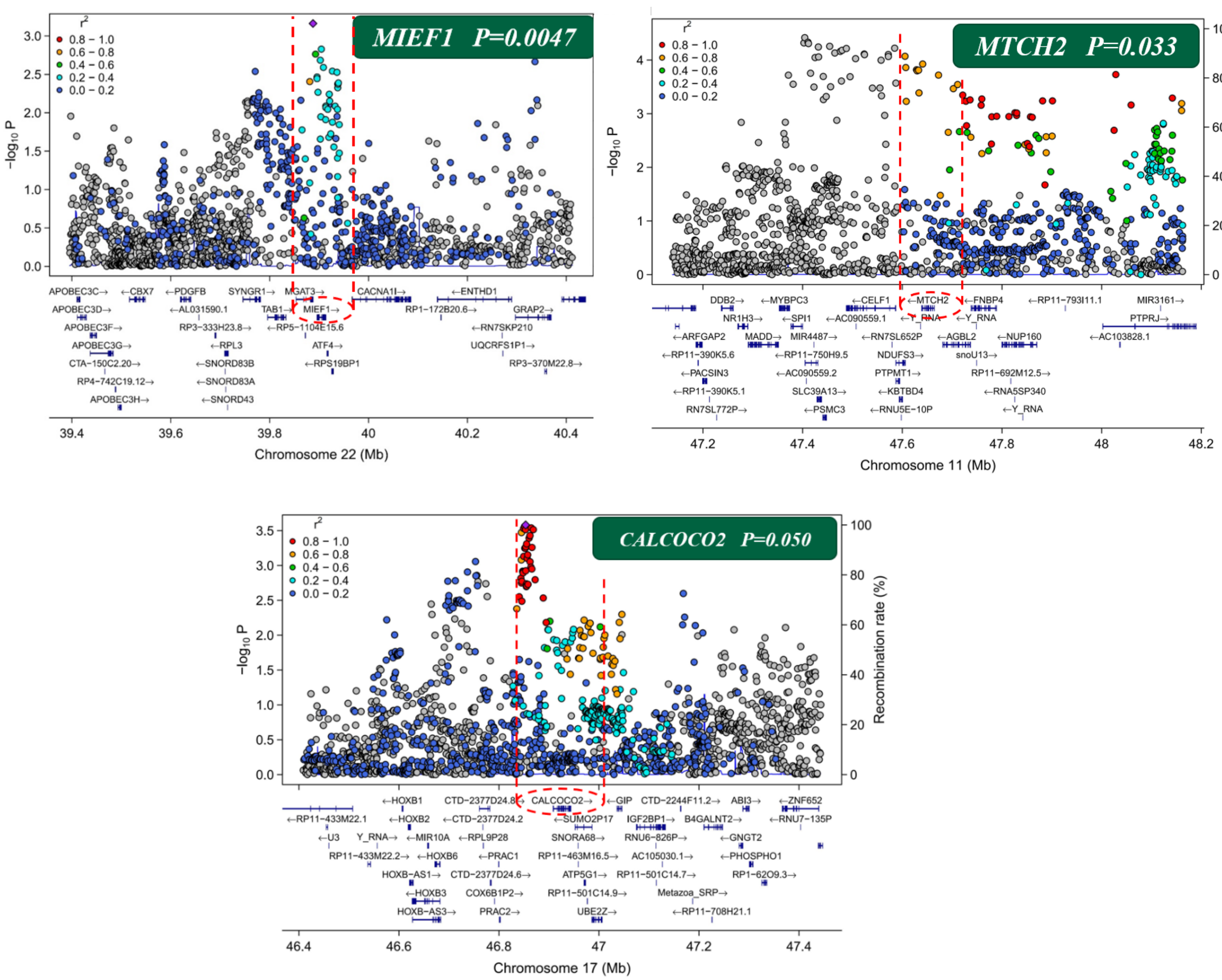

Figure S1. Additional mitochondrial genes which demonstrated nominal associations in gene-based association analysis of common variants in MTAG

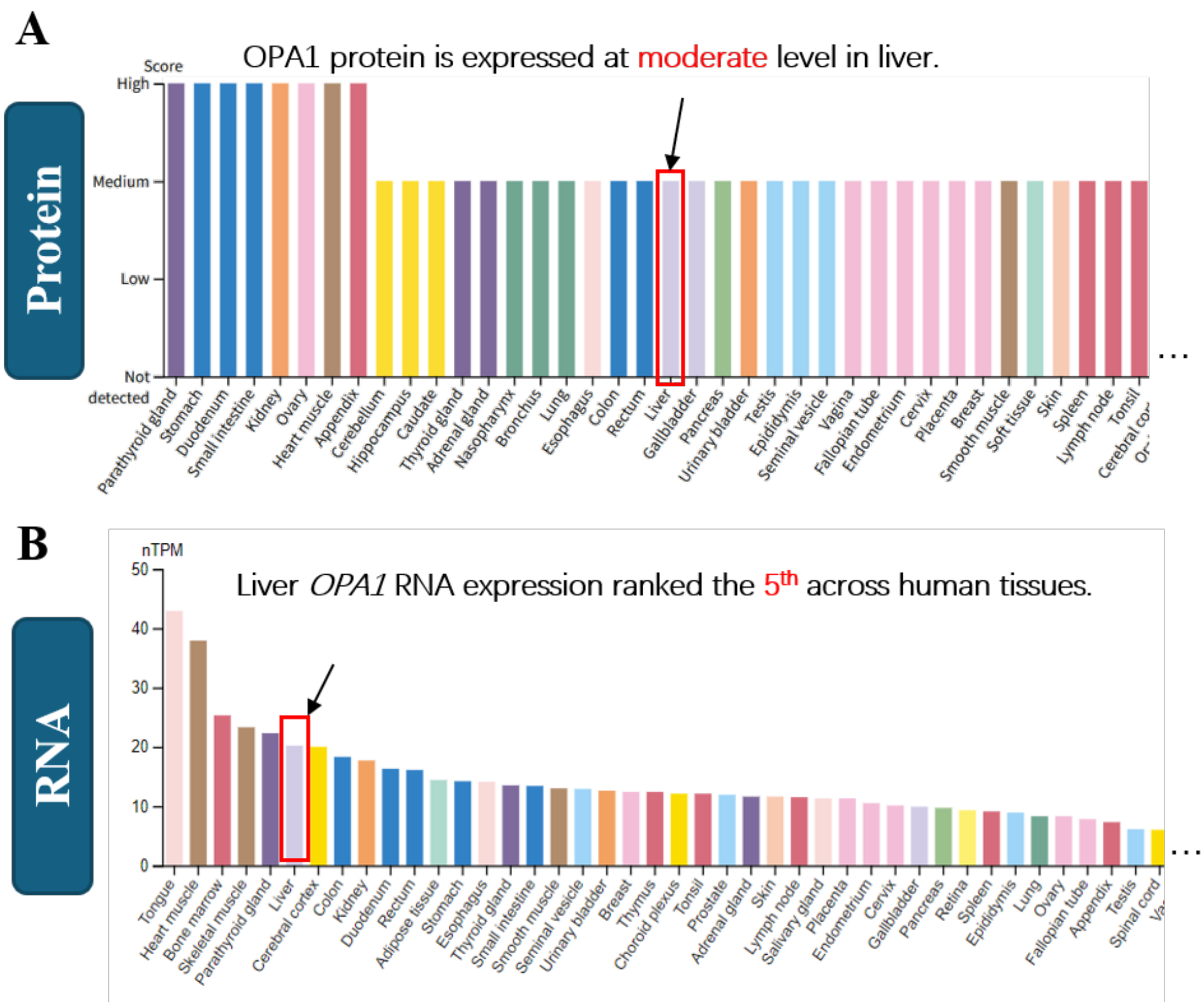

Figure S2. OPA1 protein and RNA expression across human tissues. A. The upper panel shows semiquantitative OPA1 protein expression across human tissues, categorized as high, medium, low, or not detected. Liver tissue is highlighted by a red box and arrow, indicating moderate OPA1 protein expression. B. The lower panel shows tissue-level ***OPA1*** RNA expression measured as normalized transcripts per million (nTPM). Liver is highlighted and ranks fifth among the surveyed human tissues for ***OPA1*** RNA expression, supporting prominent hepatic expression of *OPA1* at both the transcript and protein levels.

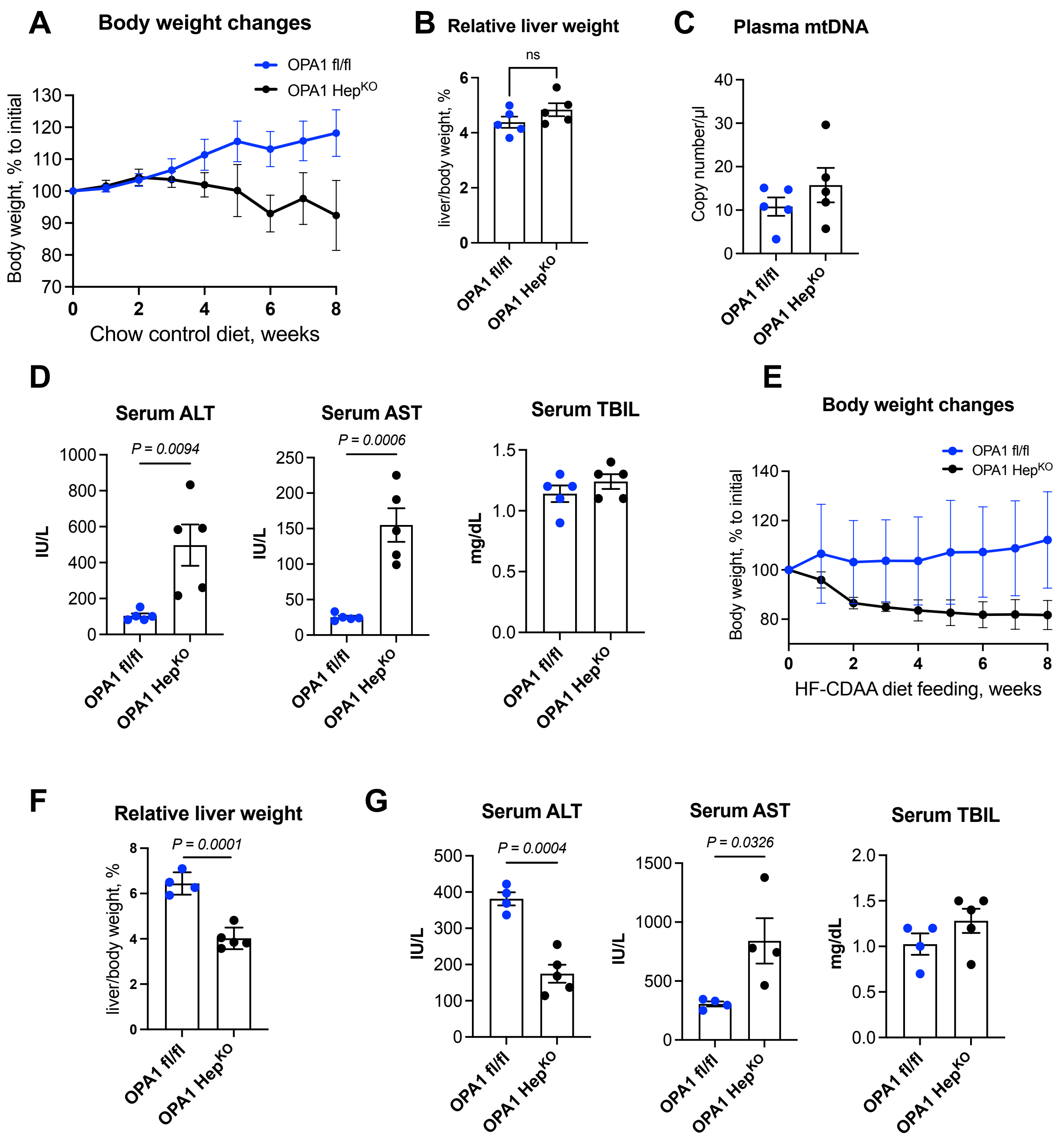

Figure S3. Body weight changes, hepatomegaly and serum chemistry upon hepatocyte-specific deletion of OPA1 in mice fed control diet and with experimental MASH. Hepatocyte-specific deletion of OPA1(OPA1 Hep^{KO}) was achieved by AAV8-TBG-Cre injection into male floxed OPA1 mice. Equivalent dose of AAV8-EGFP was used in OPA1-sufficient control group ($OPA1^{fl/fl}$). Mice were fed regular chow (A-D, n=5) or MASH diet (HF-CDAA, E-G, n=4-5) for 8 weeks before analysis. (A) Body weight changes, (B) relative liver weight, (C) circulating mtDNA measured via 12S fragment ddPCR and (D) serum ALT, AST and total bilirubin in mice fed control chow diet. (E) Body weight changes, (F) relative liver weight, and (G) serum ALT, AST and total bilirubin in mice fed control chow diet.

Table S2. Gene burden analysis of loss-of-function and missense variants in liver fibrosis and cirrhosis

			SKAT-O	SKAT	Burden
CRLS1	OPA1-pathway	LoF	0. 012	0. 008	0. 033
MICU1	OPA1-interactors	LoF	0. 012	0. 006	0. 298
BAX	OPA1-pathway	Missense	0. 013	0. 007	0. 335
PMPCA	OPA1-interactors	Missense	0. 014	1. 000	0. 006
MTX1	OPA1-pathway	Missense	0. 025	0. 271	0. 014
IMMT	OPA1-interactors	LoF	0. 027	0. 016	0. 240
IMMT	OPA1-interactors	LoF	0. 027	0. 016	0. 240
PRELID1	OPA1-interactors	LoF	0. 029	0. 019	0. 097
MICU1	OPA1-interactors	Missense	0. 084	0. 336	0. 047
HIGD1A	OPA1-interactors	LoF	0. 087	0. 063	0. 110
SAMM50	OPA1-interactors	LoF	0. 092	0. 052	0. 386
YME1L1	OPA1-interactors	LoF	0. 115	0. 089	0. 281
PMPCB	OPA1-interactors	Missense	0. 155	0. 855	0. 091
CASP3	OPA1-pathway	Missense	0. 165	0. 096	0. 197
MICOS13	OPA1-pathway	Missense	0. 167	0. 097	0. 473
SIRT3	OPA1-interactors	LoF	0. 191	0. 166	0. 130
CHCHD6	OPA1-pathway	Missense	0. 215	0. 299	0. 131
APOO	OPA1-pathway	Missense	0. 292	0. 412	0. 185
YME1L1	OPA1-interactors	Missense	0. 314	1. 000	0. 178
TAMM41	OPA1-pathway	LoF	0. 347	0. 211	0. 717
RCC1L	OPA1-interactors	Missense	0. 355	0. 204	0. 485
TRIAP1	OPA1-interactors	Missense	0. 359	0. 450	0. 243
HIGD1A	OPA1-interactors	Missense	0. 363	0. 217	0. 472
AGK	OPA1-pathway	Missense	0. 369	0. 219	0. 429
PGS1	OPA1-pathway	Missense	0. 393	0. 281	0. 248
SIRT4	OPA1-interactors	Missense	0. 403	0. 273	0. 513
AGK	OPA1-pathway	LoF	0. 409	0. 999	0. 251
PMPCB	OPA1-interactors	LoF	0. 418	0. 247	0. 715
BAK1	OPA1-pathway	Missense	0. 425	0. 999	0. 257
APAF1	OPA1-pathway	Missense	0. 469	0. 296	0. 753
CASP9	OPA1-pathway	LoF	0. 501	1. 000	0. 320
SAMM50	OPA1-interactors	Missense	0. 529	0. 593	0. 349
MTX1	OPA1-pathway	LoF	0. 575	0. 950	0. 396
ATAD3A	OPA1-interactors	LoF	0. 585	1. 000	0. 388
PGS1	OPA1-pathway	LoF	0. 601	1. 000	0. 393
PRELID1	OPA1-interactors	Missense	0. 645	0. 452	0. 702
TOMM70	OPA1-interactors	Missense	0. 670	0. 535	0. 456
PFKFB3	OPA1-interactors	Missense	0. 684	0. 511	0. 472
SIRT3	OPA1-interactors	Missense	0. 689	0. 924	0. 496
CHCHD3	OPA1-interactors	Missense	0. 707	0. 933	0. 489
CHCHD3	OPA1-interactors	Missense	0. 707	0. 933	0. 489
CASP3	OPA1-pathway	LoF	0. 714	0. 906	0. 564

			SKAT-O	SKAT	Burden
CASP9	OPA1-pathway	Missense	0. 730	0. 522	0. 711
CHCHD6	OPA1-pathway	LoF	0. 735	1. 000	0. 522
BAK1	OPA1-pathway	LoF	0. 738	1. 000	0. 543
SIRT4	OPA1-interactors	LoF	0. 742	1. 000	0. 564
CRLS1	OPA1-pathway	Missense	0. 748	0. 555	0. 586
PMPCA	OPA1-interactors	LoF	0. 751	1. 000	0. 533
IMMT	OPA1-interactors	Missense	0. 759	0. 577	0. 875
IMMT	OPA1-interactors	Missense	0. 759	0. 577	0. 875
APAF1	OPA1-pathway	LoF	0. 760	0. 535	0. 997
MTX2	OPA1-pathway	LoF	0. 809	1. 000	0. 613
MTX2	OPA1-pathway	Missense	0. 817	0. 999	0. 601
RCC1L	OPA1-interactors	LoF	0. 818	0. 603	0. 940
MICOS13	OPA1-pathway	LoF	0. 822	1. 000	0. 660
PFKFB3	OPA1-interactors	LoF	0. 876	1. 000	0. 670
NEDD4L	OPA1-interactors	LoF	0. 881	1. 000	0. 677
BAX	OPA1-pathway	LoF	0. 883	1. 000	0. 701
APOOL	OPA1-pathway	Missense	0. 886	0. 852	0. 688
TAFAZZIN	OPA1-pathway	Missense	0. 899	0. 989	0. 708
CHCHD3	OPA1-interactors	LoF	0. 903	1. 000	0. 731
CHCHD3	OPA1-interactors	LoF	0. 903	1. 000	0. 731
TOMM70	OPA1-interactors	LoF	1. 000	1. 000	0. 802
TRIAP1	OPA1-interactors	LoF	1. 000	1. 000	0. 794
APOO	OPA1-pathway	LoF	1. 000	1. 000	0. 875
APOOL	OPA1-pathway	LoF	1. 000	1. 000	0. 794
CYCS	OPA1-pathway	LoF	1. 000	1. 000	0. 888
MICOS10	OPA1-pathway	LoF	1. 000	0. 999	0. 909
TAFAZZIN	OPA1-pathway	LoF	1. 000	1. 000	0. 915
ATAD3A	OPA1-interactors	Missense	1. 000	0. 944	0. 772
NEDD4L	OPA1-interactors	Missense	1. 000	0. 844	0. 880
CYCS	OPA1-pathway	Missense	1. 000	0. 999	0. 837
MICOS10	OPA1-pathway	Missense	1. 000	0. 953	0. 816
TAMM41	OPA1-pathway	Missense	1. 000	0. 953	0. 928

Table S3. Gene burden analysis of loss-of-function and missense variants in MASLD in UK Biobank

			SKAT-O	SKAT	Burden
BAK1	OPA1-pathway	LoF	0. 013	0. 013	0. 013
CHCHD6	OPA1-pathway	LoF	0. 014	0. 008	0. 050
APOOL	OPA1-pathway	LoF	0. 017	0. 011	0. 071
CYCS	OPA1-pathway	LoF	0. 021	0. 017	0. 036
CYCS	OPA1-pathway	Missense	0. 029	0. 019	0. 042
TAFAZZIN	OPA1-pathway	Missense	0. 045	0. 082	0. 030
TRIAP1	OPA1-interactors	LoF	0. 065	0. 039	0. 207
PFKFB3	OPA1-interactors	Missense	0. 066	0. 180	0. 037
IMMT	OPA1-interactors	Missense	0. 103	0. 197	0. 062
IMMT	OPA1-interactors	Missense	0. 103	0. 197	0. 062
PMPCA	OPA1-interactors	Missense	0. 129	0. 148	0. 083
APAF1	OPA1-pathway	LoF	0. 157	0. 117	0. 118
MICU1	OPA1-interactors	LoF	0. 158	0. 874	0. 092
PRELID1	OPA1-interactors	LoF	0. 162	0. 093	0. 494
PFKFB3	OPA1-interactors	LoF	0. 181	0. 102	0. 584
APOO	OPA1-pathway	Missense	0. 186	0. 114	0. 331
PMPCB	OPA1-interactors	LoF	0. 199	0. 109	0. 235
TAMM41	OPA1-pathway	Missense	0. 213	0. 179	0. 127
BAX	OPA1-pathway	LoF	0. 219	0. 130	0. 427
AGK	OPA1-pathway	LoF	0. 223	0. 538	0. 130
HIGD1A	OPA1-interactors	Missense	0. 268	0. 999	0. 154
HIGD1A	OPA1-interactors	LoF	0. 283	0. 185	0. 720
MTX1	OPA1-pathway	Missense	0. 291	0. 217	0. 177
MICOS13	OPA1-pathway	LoF	0. 301	0. 187	0. 667
APOOL	OPA1-pathway	Missense	0. 333	0. 194	0. 951
PGS1	OPA1-pathway	Missense	0. 335	0. 307	0. 208
NEDD4L	OPA1-interactors	Missense	0. 340	0. 207	0. 682
CASP9	OPA1-pathway	Missense	0. 358	0. 635	0. 219
CRLS1	OPA1-pathway	LoF	0. 397	0. 242	0. 303
TAMM41	OPA1-pathway	LoF	0. 401	0. 244	0. 559
MTX2	OPA1-pathway	Missense	0. 417	0. 251	0. 908
TRIAP1	OPA1-interactors	Missense	0. 420	0. 894	0. 290
SAMM50	OPA1-interactors	Missense	0. 429	0. 399	0. 273
TOMM70	OPA1-interactors	Missense	0. 435	0. 267	0. 786
YME1L1	OPA1-interactors	LoF	0. 481	0. 376	0. 373
CRLS1	OPA1-pathway	Missense	0. 497	0. 372	0. 329
AGK	OPA1-pathway	Missense	0. 507	0. 314	0. 550
SIRT3	OPA1-interactors	LoF	0. 522	0. 832	0. 382
MTX2	OPA1-pathway	LoF	0. 558	1. 000	0. 369
APAF1	OPA1-pathway	Missense	0. 598	0. 397	0. 529
IMMT	OPA1-interactors	LoF	0. 626	0. 401	0. 841
IMMT	OPA1-interactors	LoF	0. 626	0. 401	0. 841
MICOS10	OPA1-pathway	Missense	0. 664	0. 482	0. 720
MICU1	OPA1-interactors	Missense	0. 689	0. 471	0. 652

			SKAT-O	SKAT	Burden
NEDD4L	OPA1-interactors	LoF	0.697	1.000	0.468
CHCHD3	OPA1-interactors	LoF	0.749	1.000	0.539
CHCHD3	OPA1-interactors	LoF	0.749	1.000	0.539
SIRT3	OPA1-interactors	Missense	0.751	0.691	0.558
CASP9	OPA1-pathway	LoF	0.792	0.848	0.589
RCC1L	OPA1-interactors	LoF	0.829	0.978	0.616
TOMM70	OPA1-interactors	LoF	0.842	1.000	0.641
MTX1	OPA1-pathway	LoF	0.860	0.715	0.897
SAMM50	OPA1-interactors	LoF	0.862	0.661	0.866
CHCHD3	OPA1-interactors	Missense	0.869	0.670	0.982
CHCHD3	OPA1-interactors	Missense	0.869	0.670	0.982
CASP3	OPA1-pathway	Missense	0.872	0.803	0.695
SIRT4	OPA1-interactors	LoF	0.879	0.725	0.955
BAX	OPA1-pathway	Missense	0.885	0.722	0.699
PMPCA	OPA1-interactors	LoF	0.898	0.894	0.727
PMPCB	OPA1-interactors	Missense	0.902	0.747	0.775
BAK1	OPA1-pathway	Missense	0.907	0.773	0.729
APOO	OPA1-pathway	LoF	0.913	1.000	0.765
RCC1L	OPA1-interactors	Missense	0.932	0.756	0.790
YME1L1	OPA1-interactors	Missense	0.936	0.767	0.959
ATAD3A	OPA1-interactors	LoF	1.000	0.898	0.820
CASP3	OPA1-pathway	LoF	1.000	0.922	0.834
MICOS10	OPA1-pathway	LoF	1.000	1.000	0.835
PGS1	OPA1-pathway	LoF	1.000	0.902	0.942
TAFAZZIN	OPA1-pathway	LoF	1.000	1.000	0.861
ATAD3A	OPA1-interactors	Missense	1.000	0.938	0.819
PRELID1	OPA1-interactors	Missense	1.000	0.989	0.819
SIRT4	OPA1-interactors	Missense	1.000	0.991	0.832
CHCHD6	OPA1-pathway	Missense	1.000	0.934	0.995
MICOS13	OPA1-pathway	Missense	1.000	0.807	0.881

Table S4. *OPA1* eQTL summary across different tissues

MTAG SNP	eQTL SNP	Position GRCH37	R^2	Tissue	Effect AF	Effect_Size	P_{eQTL}	a1	a2	N_{MTAG}	a1 frequency	Beta	P_{MTAG}
rs78912308	rs78912308	chr3:193266100	1.00	Cells - Cultured fibroblasts	A=0.101	-0.2473	6.77E-23	C	A	1621580	0.90	-0.00409	9.87E-03
rs78912308	rs78912308	chr3:193266100	1.00	Whole Blood	A=0.101	-0.1833	9.55E-07	C	A	1621580	0.90	-0.00409	9.87E-03
rs76205181	rs78912308	chr3:193273067	0.94	Whole Blood	T=0.096	-0.1787	1.70E-06	C	T	1522690	0.90	-0.0053	1.11E-03
rs76205181	rs78912308	chr3:193273067	0.94	Cells - Cultured fibroblasts	T=0.096	-0.2439	2.19E-22	C	T	1522690	0.90	-0.0053	1.11E-03
rs1872532	rs1872532	chr3:193284624	1.00	Whole Blood	C=0.328	-0.0913	6.62E-06	T	C	1800310	0.67	-0.00276	3.94E-03
rs1872532	rs1872532	chr3:193284624	1.00	Cells - Cultured fibroblasts	C=0.328	-0.0739	1.09E-06	T	C	1800310	0.67	-0.00276	3.94E-03
rs7625424	rs1872532	chr3:193285897	1.00	Cells - Cultured fibroblasts	C=0.328	-0.0744	1.06E-06	T	C	1800310	0.66	-0.0027	4.67E-03
rs7625424	rs1872532	chr3:193285897	1.00	Whole Blood	C=0.328	-0.0872	1.68E-05	T	C	1800310	0.66	-0.0027	4.67E-03
rs6444728	rs1872532	chr3:193286182	1.00	Cells - Cultured fibroblasts	T=0.328	-0.0744	1.06E-06	C	T	1624360	0.66	-0.00332	8.54E-04
rs6444728	rs1872532	chr3:193286182	1.00	Whole Blood	T=0.328	-0.0872	1.68E-05	C	T	1624360	0.66	-0.00332	8.54E-04
rs9869967	rs1872532	chr3:193287410	0.87	Whole Blood	A=0.298	-0.0896	1.79E-05	G	A	1701420	0.70	-0.00327	1.19E-03
rs9869967	rs1872532	chr3:193287410	0.87	Cells - Cultured fibroblasts	A=0.298	-0.0923	1.97E-09	G	A	1701420	0.70	-0.00327	1.19E-03
rs13326186	rs1872532	chr3:193288057	0.87	Cells - Cultured fibroblasts	A=0.298	-0.0928	1.55E-09	G	A	1800310	0.70	-0.00289	3.26E-03
rs13326186	rs1872532	chr3:193288057	0.87	Whole Blood	A=0.298	-0.0897	1.72E-05	G	A	1800310	0.70	-0.00289	3.26E-03
rs13326189	rs1872532	chr3:193288133	1.00	Cells - Cultured fibroblasts	A=0.328	-0.0744	1.06E-06	G	A	1800310	0.66	-0.00269	4.90E-03
rs13326189	rs1872532	chr3:193288133	1.00	Whole Blood	A=0.328	-0.0872	1.68E-05	G	A	1800310	0.66	-0.00269	4.90E-03
rs3863983	rs1872532	chr3:193288804	1.00	Whole Blood	C=0.328	-0.0864	2.16E-05	T	C	1624360	0.66	-0.00332	8.75E-04
rs3863983	rs1872532	chr3:193288804	1.00	Cells - Cultured fibroblasts	C=0.328	-0.0739	1.33E-06	T	C	1624360	0.66	-0.00332	8.75E-04
rs7650397	rs1872532	chr3:193290121	0.93	Cells - Cultured fibroblasts	C=0.313	-0.0778	4.44E-07	T	C	1800310	0.67	-0.00276	4.09E-03
rs7650397	rs1872532	chr3:193290121	0.93	Whole Blood	C=0.313	-0.0885	1.41E-05	T	C	1800310	0.67	-0.00276	4.09E-03
rs11922377	rs1872532	chr3:193290542	0.93	Whole Blood	G=0.313	-0.0885	1.41E-05	A	G	1645020	0.67	-0.00286	3.84E-03
rs11922377	rs1872532	chr3:193290542	0.93	Cells - Cultured fibroblasts	G=0.313	-0.0778	4.44E-07	A	G	1645020	0.67	-0.00286	3.84E-03
rs4597663	rs4597663	chr3:193291405	1.00	Whole Blood	T=0.47	-0.0988	1.51E-07	C	T	1800310	0.52	-0.0029	1.36E-03
rs4597663	rs2935303	chr3:193291405	0.70	Whole Blood	T=0.47	-0.0988	1.51E-07	C	T	1800310	0.52	-0.0029	1.36E-03
rs13067143	rs2935303	chr3:193291640	0.70	Whole Blood	T=0.47	-0.0988	1.51E-07	G	T	1701420	0.52	-0.00356	1.26E-04
rs13067143	rs4597663	chr3:193291640	1.00	Whole Blood	T=0.47	-0.0988	1.51E-07	G	T	1701420	0.52	-0.00356	1.26E-04
rs11916762	rs4597663	chr3:193292035	1.00	Whole Blood	A=0.47	-0.0988	1.51E-07	G	A	1800310	0.52	-0.00291	1.31E-03
rs11916762	rs2935303	chr3:193292035	0.70	Whole Blood	A=0.47	-0.0988	1.51E-07	G	A	1800310	0.52	-0.00291	1.31E-03

MTAG SNP	eQTL SNP	Position GRCH37	R^2	Tissue	Effect AF	Effect_Size	P_{eQTL}	a1	a2	N_{MTAG}	a1 frequency	Beta	P_{MTAG}
rs56102513	rs78912308	chr3:193292325	0.94	Cells - Cultured fibroblasts	C=0.096	-0.2395	4.92E-22	T	C	1621580	0.90	-0.0043	6.73E-03
rs56102513	rs78912308	chr3:193292325	0.94	Pancreas	C=0.096	-0.3023	3.87E-05	T	C	1621580	0.90	-0.0043	6.73E-03
rs56102513	rs78912308	chr3:193292325	0.94	Whole Blood	C=0.096	-0.1840	4.85E-07	T	C	1621580	0.90	-0.0043	6.73E-03
rs7615623	rs4597663	chr3:193293157	0.88	Cells - Cultured fibroblasts	C=0.439	-0.0680	3.06E-06	T	C	1800310	0.56	-0.00296	1.19E-03
rs7615623	rs4597663	chr3:193293157	0.88	Whole Blood	C=0.439	-0.0977	2.47E-07	T	C	1800310	0.56	-0.00296	1.19E-03
rs7626230	rs4597663	chr3:193293533	1.00	Whole Blood	C=0.47	-0.0988	1.51E-07	A	C	1701420	0.52	-0.00353	1.43E-04
rs7626230	rs2935303	chr3:193293533	0.70	Whole Blood	C=0.47	-0.0988	1.51E-07	A	C	1701420	0.52	-0.00353	1.43E-04
rs12696669	rs4597663	chr3:193293699	1.00	Whole Blood	A=0.47	-0.0988	1.51E-07	C	A	1800310	0.52	-0.0029	1.40E-03
rs12696669	rs2935303	chr3:193293699	0.70	Whole Blood	A=0.47	-0.0988	1.51E-07	C	A	1800310	0.52	-0.0029	1.40E-03
rs13324968	rs4597663	chr3:193293922	0.88	Cells - Cultured fibroblasts	G=0.439	-0.0687	2.36E-06	A	G	1800310	0.56	-0.00294	1.28E-03
rs13324968	rs4597663	chr3:193293922	0.88	Whole Blood	G=0.439	-0.0980	2.21E-07	A	G	1800310	0.56	-0.00294	1.28E-03
rs61289905	rs4597663	chr3:193295208	0.88	Cells - Cultured fibroblasts	C=0.439	-0.0680	3.06E-06	T	C	1800310	0.56	-0.00297	1.13E-03
rs61289905	rs4597663	chr3:193295208	0.88	Whole Blood	C=0.439	-0.0977	2.47E-07	T	C	1800310	0.56	-0.00297	1.13E-03
rs10804956	rs4597663	chr3:193297321	1.00	Whole Blood	C=0.47	-0.0990	1.46E-07	T	C	1800310	0.52	-0.00291	1.33E-03
rs10804956	rs2935303	chr3:193297321	0.70	Whole Blood	C=0.47	-0.0990	1.46E-07	T	C	1800310	0.52	-0.00291	1.33E-03
rs77568410	rs78912308	chr3:193297467	0.94	Pancreas	T=0.096	-0.3023	3.87E-05	C	T	1621580	0.90	-0.00432	6.48E-03
rs77568410	rs78912308	chr3:193297467	0.94	Whole Blood	T=0.096	-0.1840	4.85E-07	C	T	1621580	0.90	-0.00432	6.48E-03
rs77568410	rs78912308	chr3:193297467	0.94	Cells - Cultured fibroblasts	T=0.096	-0.2395	4.92E-22	C	T	1621580	0.90	-0.00432	6.48E-03
rs75745995	rs78912308	chr3:193298153	0.94	Cells - Cultured fibroblasts	A=0.096	-0.2395	4.92E-22	G	A	1466300	0.90	-0.00451	5.95E-03
rs75745995	rs78912308	chr3:193298153	0.94	Pancreas	A=0.096	-0.3023	3.87E-05	G	A	1466300	0.90	-0.00451	5.95E-03
rs75745995	rs78912308	chr3:193298153	0.94	Whole Blood	A=0.096	-0.1840	4.85E-07	G	A	1466300	0.90	-0.00451	5.95E-03
rs6444729	rs2935303	chr3:193298641	0.70	Whole Blood	C=0.47	-0.0990	1.46E-07	T	C	1800310	0.52	-0.0029	1.36E-03
rs6444729	rs4597663	chr3:193298641	1.00	Whole Blood	C=0.47	-0.0990	1.46E-07	T	C	1800310	0.52	-0.0029	1.36E-03
rs2134880	rs2935303	chr3:193299022	0.70	Whole Blood	C=0.47	-0.0990	1.46E-07	A	C	1800310	0.52	-0.0029	1.39E-03
rs2134880	rs4597663	chr3:193299022	1.00	Whole Blood	C=0.47	-0.0990	1.46E-07	A	C	1800310	0.52	-0.0029	1.39E-03
rs2134881	rs4597663	chr3:193299030	1.00	Whole Blood	C=0.47	-0.0990	1.46E-07	T	C	1800310	0.52	-0.00289	1.44E-03
rs2134881	rs2935303	chr3:193299030	0.70	Whole Blood	C=0.47	-0.0990	1.46E-07	T	C	1800310	0.52	-0.00289	1.44E-03
rs2174320	rs2935303	chr3:193299124	0.70	Whole Blood	A=0.47	-0.0990	1.46E-07	G	A	1800310	0.52	-0.0029	1.37E-03
rs2174320	rs4597663	chr3:193299124	1.00	Whole Blood	A=0.47	-0.0990	1.46E-07	G	A	1800310	0.52	-0.0029	1.37E-03
rs2174321	rs4597663	chr3:193299155	0.88	Cells - Cultured fibroblasts	A=0.439	-0.0680	3.06E-06	G	A	1701420	0.56	-0.00351	1.71E-04

MTAG SNP	eQTL SNP	Position GRCH37	R^2	Tissue	Effect AF	Effect_Size	P_{eQTL}	a1	a2	N_{MTAG}	a1 frequency	Beta	P_{MTAG}
rs2174321	rs4597663	chr3:193299155	0.88	Whole Blood	A=0.439	-0.0977	2.47E-07	G	A	1701420	0.56	-0.00351	1.71E-04
rs7650353	rs2935303	chr3:193299182	0.70	Whole Blood	T=0.47	-0.0990	1.46E-07	C	T	1800310	0.52	-0.00289	1.43E-03
rs7650353	rs4597663	chr3:193299182	1.00	Whole Blood	T=0.47	-0.0990	1.46E-07	C	T	1800310	0.52	-0.00289	1.43E-03
rs56002867	rs78912308	chr3:193299226	0.94	Cells - Cultured fibroblasts	T=0.096	-0.2395	4.92E-22	C	T	1621580	0.90	-0.00426	7.23E-03
rs56002867	rs78912308	chr3:193299226	0.94	Whole Blood	T=0.096	-0.1840	4.85E-07	C	T	1621580	0.90	-0.00426	7.23E-03
rs56002867	rs78912308	chr3:193299226	0.94	Pancreas	T=0.096	-0.3023	3.87E-05	C	T	1621580	0.90	-0.00426	7.23E-03
rs7650444	rs4597663	chr3:193299273	1.00	Whole Blood	T=0.47	-0.0990	1.46E-07	C	T	1645020	0.52	-0.00294	1.68E-03
rs7650444	rs2935303	chr3:193299273	0.70	Whole Blood	T=0.47	-0.0990	1.46E-07	C	T	1645020	0.52	-0.00294	1.68E-03
rs7628243	rs4597663	chr3:193299325	1.00	Whole Blood	G=0.47	-0.0990	1.46E-07	A	G	1800310	0.52	-0.00289	1.45E-03
rs7628243	rs2935303	chr3:193299325	0.70	Whole Blood	G=0.47	-0.0990	1.46E-07	A	G	1800310	0.52	-0.00289	1.45E-03
rs75547111	rs78912308	chr3:193299411	0.94	Cells - Cultured fibroblasts	C=0.096	-0.2395	4.92E-22	T	C	1800310	0.90	-0.00391	9.24E-03
rs75547111	rs78912308	chr3:193299411	0.94	Pancreas	C=0.096	-0.3023	3.87E-05	T	C	1800310	0.90	-0.00391	9.24E-03
rs75547111	rs78912308	chr3:193299411	0.94	Whole Blood	C=0.096	-0.1840	4.85E-07	T	C	1800310	0.90	-0.00391	9.24E-03
rs7620342	rs2935303	chr3:193299614	0.70	Whole Blood	G=0.47	-0.0990	1.46E-07	T	G	1800310	0.52	-0.00291	1.33E-03
rs7620342	rs4597663	chr3:193299614	1.00	Whole Blood	G=0.47	-0.0990	1.46E-07	T	G	1800310	0.52	-0.00291	1.33E-03
rs7653046	rs4597663	chr3:193299631	0.88	Whole Blood	T=0.439	-0.0977	2.47E-07	C	T	1800310	0.56	-0.00293	1.31E-03
rs7653046	rs4597663	chr3:193299631	0.88	Cells - Cultured fibroblasts	T=0.439	-0.0680	3.06E-06	C	T	1800310	0.56	-0.00293	1.31E-03
rs10937592	rs2935303	chr3:193301038	0.68	Whole Blood	A=0.475	-0.0982	1.78E-07	G	A	1624360	0.52	-0.00372	7.97E-05
rs10937592	rs4597663	chr3:193301038	0.98	Whole Blood	A=0.475	-0.0982	1.78E-07	G	A	1624360	0.52	-0.00372	7.97E-05
rs7634678	rs2935303	chr3:193301531	0.70	Whole Blood	G=0.47	-0.0990	1.46E-07	A	G	1213560	0.49	-0.00351	9.78E-04
rs7634678	rs4597663	chr3:193301531	1.00	Whole Blood	G=0.47	-0.0990	1.46E-07	A	G	1213560	0.49	-0.00351	9.78E-04
rs7627228	rs2935303	chr3:193302226	0.70	Whole Blood	G=0.47	-0.0990	1.46E-07	T	G	1800310	0.52	-0.00288	1.49E-03
rs7627228	rs4597663	chr3:193302226	1.00	Whole Blood	G=0.47	-0.0990	1.46E-07	T	G	1800310	0.52	-0.00288	1.49E-03
rs9846719	rs2935303	chr3:193302720	0.70	Whole Blood	T=0.47	-0.0985	1.59E-07	C	T	1800310	0.53	-0.00278	2.13E-03
rs9846719	rs4597663	chr3:193302720	1.00	Whole Blood	T=0.47	-0.0985	1.59E-07	C	T	1800310	0.53	-0.00278	2.13E-03
rs34023161	rs2935303	chr3:193302752	0.70	Whole Blood	T=0.47	-0.0985	1.59E-07	C	T	1392290	0.53	-0.00357	3.34E-04
rs34023161	rs4597663	chr3:193302752	1.00	Whole Blood	T=0.47	-0.0985	1.59E-07	C	T	1392290	0.53	-0.00357	3.34E-04
rs7618459	rs4597663	chr3:193303032	0.88	Whole Blood	T=0.439	-0.0977	2.47E-07	C	T	1701420	0.56	-0.00351	1.72E-04
rs7618459	rs4597663	chr3:193303032	0.88	Cells - Cultured fibroblasts	T=0.439	-0.0680	3.06E-06	C	T	1701420	0.56	-0.00351	1.72E-04
rs7618541	rs4597663	chr3:193303101	0.88	Cells - Cultured fibroblasts	T=0.439	-0.0683	2.92E-06	C	T	1723250	0.55	-0.00318	5.74E-04

MTAG SNP	eQTL SNP	Position GRCH37	R^2	Tissue	Effect AF	Effect_Size	P_{eQTL}	a1	a2	N_{MTAG}	a1 frequency	Beta	P_{MTAG}
rs7618541	rs4597663	chr3:193303101	0.88	Whole Blood	T=0.439	-0.0980	2.34E-07	C	T	1723250	0.55	-0.00318	5.74E-04
rs13064790	rs4597663	chr3:193303535	0.88	Whole Blood	A=0.439	-0.0987	1.88E-07	G	A	1800310	0.56	-0.00297	1.11E-03
rs13064790	rs4597663	chr3:193303535	0.88	Cells - Cultured fibroblasts	A=0.439	-0.0678	3.58E-06	G	A	1800310	0.56	-0.00297	1.11E-03
rs9856156	rs4597663	chr3:193314595	1.00	Whole Blood	A=0.47	-0.1020	6.64E-08	G	A	1701420	0.53	-0.00363	9.26E-05
rs9856156	rs2935303	chr3:193314595	0.70	Cells - Cultured fibroblasts	A=0.47	-0.0586	7.01E-05	G	A	1701420	0.53	-0.00363	9.26E-05
rs9856156	rs4597663	chr3:193314595	1.00	Cells - Cultured fibroblasts	A=0.47	-0.0586	7.01E-05	G	A	1701420	0.53	-0.00363	9.26E-05
rs9856156	rs2935303	chr3:193314595	0.70	Whole Blood	A=0.47	-0.1020	6.64E-08	G	A	1701420	0.53	-0.00363	9.26E-05
rs75977202	rs78912308	chr3:193315176	0.94	Cells - Cultured fibroblasts	A=0.096	-0.2407	2.90E-22	G	A	1621580	0.90	-0.00455	4.23E-03
rs75977202	rs78912308	chr3:193315176	0.94	Whole Blood	A=0.096	-0.1849	4.79E-07	G	A	1621580	0.90	-0.00455	4.23E-03
rs75977202	rs78912308	chr3:193315176	0.94	Pancreas	A=0.096	-0.3050	3.79E-05	G	A	1621580	0.90	-0.00455	4.23E-03
rs4263236	rs4597663	chr3:193315331	1.00	Cells - Cultured fibroblasts	T=0.47	-0.0607	4.86E-05	G	T	1800310	0.53	-0.00301	9.02E-04
rs4263236	rs2935303	chr3:193315331	0.70	Whole Blood	T=0.47	-0.1006	1.08E-07	G	T	1800310	0.53	-0.00301	9.02E-04
rs4263236	rs4597663	chr3:193315331	1.00	Whole Blood	T=0.47	-0.1006	1.08E-07	G	T	1800310	0.53	-0.00301	9.02E-04
rs4263236	rs2935303	chr3:193315331	0.70	Cells - Cultured fibroblasts	T=0.47	-0.0607	4.86E-05	G	T	1800310	0.53	-0.00301	9.02E-04
rs12696670	rs4597663	chr3:193316048	0.88	Cells - Cultured fibroblasts	G=0.439	-0.0676	3.62E-06	A	G	1800310	0.56	-0.00291	1.43E-03
rs12696670	rs4597663	chr3:193316048	0.88	Whole Blood	G=0.439	-0.0973	2.51E-07	A	G	1800310	0.56	-0.00291	1.43E-03
rs6800240	rs2935303	chr3:193318250	0.70	Whole Blood	C=0.47	-0.1036	4.26E-08	T	C	1800310	0.52	-0.00291	1.31E-03
rs6800240	rs2935303	chr3:193318250	0.70	Cells - Cultured fibroblasts	C=0.47	-0.0606	4.30E-05	T	C	1800310	0.52	-0.00291	1.31E-03
rs6800240	rs4597663	chr3:193318250	1.00	Whole Blood	C=0.47	-0.1036	4.26E-08	T	C	1800310	0.52	-0.00291	1.31E-03
rs6800240	rs4597663	chr3:193318250	1.00	Cells - Cultured fibroblasts	C=0.47	-0.0606	4.30E-05	T	C	1800310	0.52	-0.00291	1.31E-03
rs6790120	rs4597663	chr3:193318576	0.88	Whole Blood	T=0.439	-0.0973	2.51E-07	C	T	1800310	0.56	-0.00292	1.34E-03
rs6790120	rs4597663	chr3:193318576	0.88	Cells - Cultured fibroblasts	T=0.439	-0.0676	3.62E-06	C	T	1800310	0.56	-0.00292	1.34E-03
rs6794338	rs2935303	chr3:193320149	0.70	Whole Blood	A=0.47	-0.1036	4.26E-08	G	A	1701420	0.53	-0.00361	1.02E-04
rs6794338	rs4597663	chr3:193320149	1.00	Whole Blood	A=0.47	-0.1036	4.26E-08	G	A	1701420	0.53	-0.00361	1.02E-04
rs6794338	rs4597663	chr3:193320149	1.00	Cells - Cultured fibroblasts	A=0.47	-0.0606	4.30E-05	G	A	1701420	0.53	-0.00361	1.02E-04
rs6794338	rs2935303	chr3:193320149	0.70	Cells - Cultured fibroblasts	A=0.47	-0.0606	4.30E-05	G	A	1701420	0.53	-0.00361	1.02E-04
rs7619129	rs4597663	chr3:193320736	0.88	Cells - Cultured fibroblasts	G=0.439	-0.0676	3.62E-06	A	G	1701420	0.56	-0.00351	1.71E-04
rs7619129	rs4597663	chr3:193320736	0.88	Whole Blood	G=0.439	-0.0973	2.51E-07	A	G	1701420	0.56	-0.00351	1.71E-04
rs11925699	rs2935303	chr3:193321115	0.70	Whole Blood	A=0.47	-0.1059	2.35E-08	G	A	1800310	0.53	-0.00273	2.58E-03
rs11925699	rs2935303	chr3:193321115	0.70	Cells - Cultured fibroblasts	A=0.47	-0.0588	7.60E-05	G	A	1800310	0.53	-0.00273	2.58E-03

MTAG SNP	eQTL SNP	Position GRCH37	R^2	Tissue	Effect AF	Effect_Size	P_{eQTL}	a1	a2	N_{MTAG}	a1 frequency	Beta	P_{MTAG}
rs11925699	rs4597663	chr3:193321115	1.00	Whole Blood	A=0.47	-0.1059	2.35E-08	G	A	1800310	0.53	-0.00273	2.58E-03
rs11925699	rs4597663	chr3:193321115	1.00	Cells - Cultured fibroblasts	A=0.47	-0.0588	7.60E-05	G	A	1800310	0.53	-0.00273	2.58E-03
rs6797738	rs4597663	chr3:193321173	0.88	Cells - Cultured fibroblasts	A=0.439	-0.0676	3.62E-06	G	A	1800310	0.56	-0.00294	1.27E-03
rs6797738	rs4597663	chr3:193321173	0.88	Whole Blood	A=0.439	-0.0973	2.51E-07	G	A	1800310	0.56	-0.00294	1.27E-03
rs9819038	rs4597663	chr3:193322870	0.88	Cells - Cultured fibroblasts	G=0.439	-0.0676	3.62E-06	T	G	1800310	0.56	-0.00291	1.39E-03
rs9819038	rs4597663	chr3:193322870	0.88	Whole Blood	G=0.439	-0.0973	2.51E-07	T	G	1800310	0.56	-0.00291	1.39E-03
rs7613578	rs4597663	chr3:193326458	0.98	Cells - Cultured fibroblasts	T=0.475	-0.0606	4.30E-05	C	T	1491190	0.53	-0.0029	2.69E-03
rs7613578	rs4597663	chr3:193326458	0.98	Whole Blood	T=0.475	-0.1036	4.26E-08	C	T	1491190	0.53	-0.0029	2.69E-03
rs7613578	rs2935303	chr3:193326458	0.68	Cells - Cultured fibroblasts	T=0.475	-0.0606	4.30E-05	C	T	1491190	0.53	-0.0029	2.69E-03
rs7613578	rs2935303	chr3:193326458	0.68	Whole Blood	T=0.475	-0.1036	4.26E-08	C	T	1491190	0.53	-0.0029	2.69E-03
rs2101340	rs4597663	chr3:193328895	0.88	Whole Blood	G=0.439	-0.0973	2.51E-07	A	G	1800310	0.56	-0.00289	1.54E-03
rs2101340	rs4597663	chr3:193328895	0.88	Cells - Cultured fibroblasts	G=0.439	-0.0676	3.62E-06	A	G	1800310	0.56	-0.00289	1.54E-03
rs13075244	rs4597663	chr3:193329300	1.00	Cells - Cultured fibroblasts	C=0.47	-0.0586	7.01E-05	T	C	1800310	0.52	-0.00287	1.54E-03
rs13075244	rs4597663	chr3:193329300	1.00	Whole Blood	C=0.47	-0.1020	6.64E-08	T	C	1800310	0.52	-0.00287	1.54E-03
rs13075244	rs2935303	chr3:193329300	0.70	Cells - Cultured fibroblasts	C=0.47	-0.0586	7.01E-05	T	C	1800310	0.52	-0.00287	1.54E-03
rs13075244	rs2935303	chr3:193329300	0.70	Whole Blood	C=0.47	-0.1020	6.64E-08	T	C	1800310	0.52	-0.00287	1.54E-03
rs13095315	rs2935303	chr3:193329318	0.70	Whole Blood	G=0.47	-0.1036	4.26E-08	A	G	1800310	0.52	-0.00281	1.95E-03
rs13095315	rs4597663	chr3:193329318	1.00	Cells - Cultured fibroblasts	G=0.47	-0.0606	4.30E-05	A	G	1800310	0.52	-0.00281	1.95E-03
rs13095315	rs4597663	chr3:193329318	1.00	Whole Blood	G=0.47	-0.1036	4.26E-08	A	G	1800310	0.52	-0.00281	1.95E-03
rs13095315	rs2935303	chr3:193329318	0.70	Cells - Cultured fibroblasts	G=0.47	-0.0606	4.30E-05	A	G	1800310	0.52	-0.00281	1.95E-03
rs9828315	rs4597663	chr3:193330022	1.00	Whole Blood	G=0.47	-0.1036	4.26E-08	T	G	1800310	0.52	-0.0029	1.39E-03
rs9828315	rs2935303	chr3:193330022	0.70	Whole Blood	G=0.47	-0.1036	4.26E-08	T	G	1800310	0.52	-0.0029	1.39E-03
rs9828315	rs4597663	chr3:193330022	1.00	Cells - Cultured fibroblasts	G=0.47	-0.0606	4.30E-05	T	G	1800310	0.52	-0.0029	1.39E-03
rs9828315	rs2935303	chr3:193330022	0.70	Cells - Cultured fibroblasts	G=0.47	-0.0606	4.30E-05	T	G	1800310	0.52	-0.0029	1.39E-03
rs6444732	rs2935303	chr3:193330118	0.70	Whole Blood	G=0.47	-0.1013	9.45E-08	T	G	1800310	0.53	-0.00296	1.08E-03
rs6444732	rs2935303	chr3:193330118	0.70	Cells - Cultured fibroblasts	G=0.47	-0.0596	6.73E-05	T	G	1800310	0.53	-0.00296	1.08E-03
rs6444732	rs4597663	chr3:193330118	1.00	Cells - Cultured fibroblasts	G=0.47	-0.0596	6.73E-05	T	G	1800310	0.53	-0.00296	1.08E-03
rs6444732	rs4597663	chr3:193330118	1.00	Whole Blood	G=0.47	-0.1013	9.45E-08	T	G	1800310	0.53	-0.00296	1.08E-03
rs7646539	rs4597663	chr3:193334846	0.88	Whole Blood	G=0.439	-0.0982	1.83E-07	A	G	1800310	0.56	-0.00287	1.67E-03
rs7646539	rs4597663	chr3:193334846	0.88	Cells - Cultured fibroblasts	G=0.439	-0.0671	4.13E-06	A	G	1800310	0.56	-0.00287	1.67E-03

MTAG SNP	eQTL SNP	Position GRCH37	R^2	Tissue	Effect AF	Effect_Size	P_{eQTL}	a1	a2	N_{MTAG}	a1 frequency	Beta	P_{MTAG}
rs7624750	rs4597663	chr3:193334991	1.00	Whole Blood	A=0.47	-0.1045	3.17E-08	G	A	1800310	0.52	-0.00294	1.18E-03
rs7624750	rs2935303	chr3:193334991	0.70	Whole Blood	A=0.47	-0.1045	3.17E-08	G	A	1800310	0.52	-0.00294	1.18E-03
rs7624750	rs4597663	chr3:193334991	1.00	Cells - Cultured fibroblasts	A=0.47	-0.0606	4.30E-05	G	A	1800310	0.52	-0.00294	1.18E-03
rs7624750	rs2935303	chr3:193334991	0.70	Cells - Cultured fibroblasts	A=0.47	-0.0606	4.30E-05	G	A	1800310	0.52	-0.00294	1.18E-03
rs10937593	rs4597663	chr3:193335252	1.00	Whole Blood	T=0.47	-0.1045	3.17E-08	G	T	1800310	0.52	-0.0029	1.38E-03
rs10937593	rs4597663	chr3:193335252	1.00	Cells - Cultured fibroblasts	T=0.47	-0.0606	4.30E-05	G	T	1800310	0.52	-0.0029	1.38E-03
rs10937593	rs2935303	chr3:193335252	0.70	Whole Blood	T=0.47	-0.1045	3.17E-08	G	T	1800310	0.52	-0.0029	1.38E-03
rs10937593	rs2935303	chr3:193335252	0.70	Cells - Cultured fibroblasts	T=0.47	-0.0606	4.30E-05	G	T	1800310	0.52	-0.0029	1.38E-03
rs9832709	rs4597663	chr3:193336425	0.88	Cells - Cultured fibroblasts	C=0.439	-0.0676	3.62E-06	T	C	1800310	0.56	-0.0029	1.47E-03
rs9832709	rs4597663	chr3:193336425	0.88	Whole Blood	C=0.439	-0.0982	1.91E-07	T	C	1800310	0.56	-0.0029	1.47E-03
rs3772393	rs4597663	chr3:193336639	0.88	Whole Blood	C=0.439	-0.0982	1.91E-07	T	C	1701420	0.56	-0.0035	1.81E-04
rs3772393	rs4597663	chr3:193336639	0.88	Cells - Cultured fibroblasts	C=0.439	-0.0676	3.62E-06	T	C	1701420	0.56	-0.0035	1.81E-04
rs9817704	rs4597663	chr3:193337021	1.00	Cells - Cultured fibroblasts	A=0.47	-0.0606	4.30E-05	G	A	1800310	0.52	-0.00289	1.41E-03
rs9817704	rs2935303	chr3:193337021	0.70	Cells - Cultured fibroblasts	A=0.47	-0.0606	4.30E-05	G	A	1800310	0.52	-0.00289	1.41E-03
rs9817704	rs2935303	chr3:193337021	0.70	Whole Blood	A=0.47	-0.1045	3.17E-08	G	A	1800310	0.52	-0.00289	1.41E-03
rs9817704	rs4597663	chr3:193337021	1.00	Whole Blood	A=0.47	-0.1045	3.17E-08	G	A	1800310	0.52	-0.00289	1.41E-03
rs6770913	rs2935303	chr3:193338335	0.70	Whole Blood	A=0.47	-0.1036	4.26E-08	C	A	1800310	0.52	-0.00288	1.46E-03
rs6770913	rs4597663	chr3:193338335	1.00	Whole Blood	A=0.47	-0.1036	4.26E-08	C	A	1800310	0.52	-0.00288	1.46E-03
rs6770913	rs4597663	chr3:193338335	1.00	Cells - Cultured fibroblasts	A=0.47	-0.0606	4.30E-05	C	A	1800310	0.52	-0.00288	1.46E-03
rs6770913	rs2935303	chr3:193338335	0.70	Cells - Cultured fibroblasts	A=0.47	-0.0606	4.30E-05	C	A	1800310	0.52	-0.00288	1.46E-03
rs34520293	rs4597663	chr3:193340310	1.00	Whole Blood	C=0.47	-0.1036	4.26E-08	A	C	1701420	0.53	-0.00356	1.27E-04
rs34520293	rs2935303	chr3:193340310	0.70	Whole Blood	C=0.47	-0.1036	4.26E-08	A	C	1701420	0.53	-0.00356	1.27E-04
rs34520293	rs4597663	chr3:193340310	1.00	Cells - Cultured fibroblasts	C=0.47	-0.0606	4.30E-05	A	C	1701420	0.53	-0.00356	1.27E-04
rs34520293	rs2935303	chr3:193340310	0.70	Cells - Cultured fibroblasts	C=0.47	-0.0606	4.30E-05	A	C	1701420	0.53	-0.00356	1.27E-04
rs12490391	rs4597663	chr3:193341016	0.88	Cells - Cultured fibroblasts	T=0.439	-0.0676	3.62E-06	C	T	1800310	0.56	-0.00286	1.70E-03
rs12490391	rs4597663	chr3:193341016	0.88	Whole Blood	T=0.439	-0.0973	2.51E-07	C	T	1800310	0.56	-0.00286	1.70E-03
rs6444733	rs2935303	chr3:193346648	0.70	Whole Blood	C=0.47	-0.1010	8.37E-08	A	C	1701420	0.53	-0.00358	1.19E-04
rs6444733	rs4597663	chr3:193346648	1.00	Whole Blood	C=0.47	-0.1010	8.37E-08	A	C	1701420	0.53	-0.00358	1.19E-04
rs6444734	rs4597663	chr3:193346921	0.88	Cells - Cultured fibroblasts	T=0.439	-0.0676	3.62E-06	C	T	1445630	0.53	-0.00352	4.49E-04
rs6444734	rs4597663	chr3:193346921	0.88	Whole Blood	T=0.439	-0.0973	2.51E-07	C	T	1445630	0.53	-0.00352	4.49E-04

MTAG SNP	eQTL SNP	Position GRCH37	R^2	Tissue	Effect AF	Effect_Size	P_{eQTL}	a1	a2	N_{MTAG}	a1 frequency	Beta	P_{MTAG}
rs12696671	rs4597663	chr3:193348919	0.88	Cells - Cultured fibroblasts	G=0.439	-0.0676	3.62E-06	A	G	1800310	0.56	-0.00286	1.74E-03
rs12696671	rs4597663	chr3:193348919	0.88	Whole Blood	G=0.439	-0.0973	2.51E-07	A	G	1800310	0.56	-0.00286	1.74E-03
rs2101341	rs2935303	chr3:193350294	0.70	Cells - Cultured fibroblasts	C=0.47	-0.0589	6.94E-05	T	C	1800310	0.52	-0.00291	1.34E-03
rs2101341	rs2935303	chr3:193350294	0.70	Whole Blood	C=0.47	-0.1059	1.83E-08	T	C	1800310	0.52	-0.00291	1.34E-03
rs2101341	rs4597663	chr3:193350294	1.00	Whole Blood	C=0.47	-0.1059	1.83E-08	T	C	1800310	0.52	-0.00291	1.34E-03
rs2101341	rs4597663	chr3:193350294	1.00	Cells - Cultured fibroblasts	C=0.47	-0.0589	6.94E-05	T	C	1800310	0.52	-0.00291	1.34E-03
rs13096511	rs4597663	chr3:193350837	0.87	Cells - Cultured fibroblasts	G=0.434	-0.0676	3.62E-06	T	G	1723250	0.56	-0.00304	1.01E-03
rs13096511	rs4597663	chr3:193350837	0.87	Whole Blood	G=0.434	-0.0973	2.51E-07	T	G	1723250	0.56	-0.00304	1.01E-03
rs13078398	rs4597663	chr3:193350876	0.88	Whole Blood	T=0.439	-0.0973	2.51E-07	G	T	1800310	0.56	-0.00285	1.78E-03
rs13078398	rs4597663	chr3:193350876	0.88	Cells - Cultured fibroblasts	T=0.439	-0.0676	3.62E-06	G	T	1800310	0.56	-0.00285	1.78E-03
rs3736198	rs2935303	chr3:193353072	0.70	Whole Blood	A=0.47	-0.1036	4.26E-08	G	A	1701420	0.52	-0.00365	8.65E-05
rs3736198	rs4597663	chr3:193353072	1.00	Whole Blood	A=0.47	-0.1036	4.26E-08	G	A	1701420	0.52	-0.00365	8.65E-05
rs3736198	rs2935303	chr3:193353072	0.70	Cells - Cultured fibroblasts	A=0.47	-0.0606	4.30E-05	G	A	1701420	0.52	-0.00365	8.65E-05
rs3736198	rs4597663	chr3:193353072	1.00	Cells - Cultured fibroblasts	A=0.47	-0.0606	4.30E-05	G	A	1701420	0.52	-0.00365	8.65E-05
rs6807435	rs4597663	chr3:193353955	1.00	Cells - Cultured fibroblasts	A=0.47	-0.0606	4.30E-05	G	A	1800310	0.53	-0.0029	1.40E-03
rs6807435	rs2935303	chr3:193353955	0.70	Cells - Cultured fibroblasts	A=0.47	-0.0606	4.30E-05	G	A	1800310	0.53	-0.0029	1.40E-03
rs6807435	rs2935303	chr3:193353955	0.70	Whole Blood	A=0.47	-0.1047	2.84E-08	G	A	1800310	0.53	-0.0029	1.40E-03
rs6807435	rs4597663	chr3:193353955	1.00	Whole Blood	A=0.47	-0.1047	2.84E-08	G	A	1800310	0.53	-0.0029	1.40E-03
rs78386430	rs78912308	chr3:193356770	0.94	Cells - Cultured fibroblasts	G=0.096	-0.2416	5.34E-22	A	G	1522690	0.90	-0.00556	6.27E-04
rs78386430	rs78912308	chr3:193356770	0.94	Whole Blood	G=0.096	-0.1830	9.05E-07	A	G	1522690	0.90	-0.00556	6.27E-04
rs1078932	rs4597663	chr3:193358672	0.88	Cells - Cultured fibroblasts	A=0.439	-0.0676	3.62E-06	G	A	1800310	0.56	-0.00282	1.96E-03
rs1078932	rs4597663	chr3:193358672	0.88	Whole Blood	A=0.439	-0.0973	2.51E-07	G	A	1800310	0.56	-0.00282	1.96E-03
rs36146795	rs4597663	chr3:193359193	0.87	Whole Blood	C=0.434	-0.0973	2.51E-07	T	C	1723250	0.56	-0.00303	1.08E-03
rs36146795	rs4597663	chr3:193359193	0.87	Cells - Cultured fibroblasts	C=0.434	-0.0699	1.63E-06	T	C	1723250	0.56	-0.00303	1.08E-03
rs7630464	rs1872532	chr3:193359565	0.93	Cells - Cultured fibroblasts	G=0.313	-0.0864	3.48E-08	A	G	1800310	0.67	-0.00293	2.36E-03
rs7630464	rs1872532	chr3:193359565	0.93	Whole Blood	G=0.313	-0.0933	6.51E-06	A	G	1800310	0.67	-0.00293	2.36E-03
rs1016226	rs4597663	chr3:193361617	0.87	Cells - Cultured fibroblasts	T=0.434	-0.0680	3.04E-06	C	T	1800310	0.56	-0.00269	3.24E-03
rs1016226	rs4597663	chr3:193361617	0.87	Whole Blood	T=0.434	-0.0985	1.88E-07	C	T	1800310	0.56	-0.00269	3.24E-03
rs77303706	rs78912308	chr3:193361917	0.94	Cells - Cultured fibroblasts	A=0.096	-0.2416	5.34E-22	G	A	1621580	0.90	-0.00451	4.64E-03
rs77303706	rs78912308	chr3:193361917	0.94	Whole Blood	A=0.096	-0.1830	9.05E-07	G	A	1621580	0.90	-0.00451	4.64E-03

MTAG SNP	eQTL SNP	Position GRCH37	R^2	Tissue	Effect AF	Effect_Size	P_{eQTL}	a1	a2	N_{MTAG}	a1 frequency	Beta	P_{MTAG}
rs11916478	rs4597663	chr3:193362189	0.87	Whole Blood	A=0.434	-0.0957	4.08E-07	G	A	1800310	0.56	-0.00275	2.56E-03
rs11916478	rs4597663	chr3:193362189	0.87	Cells - Cultured fibroblasts	A=0.434	-0.0703	1.34E-06	G	A	1800310	0.56	-0.00275	2.56E-03
rs6771174	rs1872532	chr3:193362666	0.81	Whole Blood	T=0.283	-0.0871	4.21E-05	C	T	1800310	0.71	-0.00299	2.69E-03
rs6771174	rs1872532	chr3:193362666	0.81	Cells - Cultured fibroblasts	T=0.283	-0.0972	5.74E-10	C	T	1800310	0.71	-0.00299	2.69E-03
rs2291373	rs4597663	chr3:193363163	0.96	Cells - Cultured fibroblasts	A=0.47	-0.0596	5.91E-05	G	A	1800310	0.53	-0.00275	2.45E-03
rs2291373	rs2935303	chr3:193363163	0.73	Cells - Cultured fibroblasts	A=0.47	-0.0596	5.91E-05	G	A	1800310	0.53	-0.00275	2.45E-03
rs2291373	rs4597663	chr3:193363163	0.96	Whole Blood	A=0.47	-0.0979	2.52E-07	G	A	1800310	0.53	-0.00275	2.45E-03
rs2291373	rs2935303	chr3:193363163	0.73	Whole Blood	A=0.47	-0.0979	2.52E-07	G	A	1800310	0.53	-0.00275	2.45E-03
rs77539393	rs78912308	chr3:193363267	0.94	Whole Blood	C=0.096	-0.1838	4.66E-07	T	C	1621580	0.90	-0.0045	4.69E-03
rs77539393	rs78912308	chr3:193363267	0.94	Cells - Cultured fibroblasts	C=0.096	-0.2426	6.82E-23	T	C	1621580	0.90	-0.0045	4.69E-03
rs7621979	rs4597663	chr3:193364483	0.86	Whole Blood	T=0.444	-0.0952	4.86E-07	G	T	1645020	0.56	-0.00295	1.68E-03
rs7621979	rs4597663	chr3:193364483	0.86	Cells - Cultured fibroblasts	T=0.444	-0.0665	5.23E-06	G	T	1645020	0.56	-0.00295	1.68E-03
rs9845217	rs4597663	chr3:193365544	0.98	Whole Blood	G=0.475	-0.0973	2.76E-07	A	G	1800310	0.53	-0.00286	1.61E-03
rs9845217	rs4597663	chr3:193365544	0.98	Cells - Cultured fibroblasts	G=0.475	-0.0576	1.07E-04	A	G	1800310	0.53	-0.00286	1.61E-03
rs9845217	rs2935303	chr3:193365544	0.71	Whole Blood	G=0.475	-0.0973	2.76E-07	A	G	1800310	0.53	-0.00286	1.61E-03
rs9845217	rs2935303	chr3:193365544	0.71	Cells - Cultured fibroblasts	G=0.475	-0.0576	1.07E-04	A	G	1800310	0.53	-0.00286	1.61E-03
rs9831900	rs2935303	chr3:193365974	0.71	Cells - Cultured fibroblasts	G=0.475	-0.0610	3.76E-05	T	G	1213560	0.49	-0.00356	8.12E-04
rs9831900	rs4597663	chr3:193365974	0.98	Cells - Cultured fibroblasts	G=0.475	-0.0610	3.76E-05	T	G	1213560	0.49	-0.00356	8.12E-04
rs9831900	rs2935303	chr3:193365974	0.71	Whole Blood	G=0.475	-0.1034	4.24E-08	T	G	1213560	0.49	-0.00356	8.12E-04
rs9831900	rs4597663	chr3:193365974	0.98	Whole Blood	G=0.475	-0.1034	4.24E-08	T	G	1213560	0.49	-0.00356	8.12E-04
rs7620116	rs4597663	chr3:193368692	0.85	Cells - Cultured fibroblasts	A=0.439	-0.0669	4.39E-06	C	A	1701420	0.56	-0.00333	3.69E-04
rs7620116	rs4597663	chr3:193368692	0.85	Whole Blood	A=0.439	-0.0964	3.67E-07	C	A	1701420	0.56	-0.00333	3.69E-04
rs7620272	rs4597663	chr3:193368700	0.85	Whole Blood	A=0.439	-0.0964	3.67E-07	G	A	1800310	0.56	-0.00271	3.01E-03
rs7620272	rs4597663	chr3:193368700	0.85	Cells - Cultured fibroblasts	A=0.439	-0.0669	4.39E-06	G	A	1800310	0.56	-0.00271	3.01E-03
rs11717605	rs4597663	chr3:193369463	0.85	Whole Blood	G=0.439	-0.0952	4.86E-07	A	G	1800310	0.56	-0.00286	1.74E-03
rs11717605	rs4597663	chr3:193369463	0.85	Cells - Cultured fibroblasts	G=0.439	-0.0669	4.39E-06	A	G	1800310	0.56	-0.00286	1.74E-03
rs56263480	rs78912308	chr3:193372027	0.94	Whole Blood	A=0.096	-0.1849	4.79E-07	G	A	1621580	0.90	-0.00449	4.87E-03
rs56263480	rs78912308	chr3:193372027	0.94	Cells - Cultured fibroblasts	A=0.096	-0.2407	2.90E-22	G	A	1621580	0.90	-0.00449	4.87E-03
rs56263480	rs78912308	chr3:193372027	0.94	Pancreas	A=0.096	-0.3050	3.79E-05	G	A	1621580	0.90	-0.00449	4.87E-03
rs79129003	rs78912308	chr3:193373936	0.94	Pancreas	A=0.096	-0.3050	3.79E-05	G	A	1621580	0.90	-0.00444	5.30E-03

MTAG SNP	eQTL SNP	Position GRCH37	R^2	Tissue	Effect AF	Effect_Size	P_{eQTL}	a1	a2	N_{MTAG}	a1 frequency	Beta	P_{MTAG}
rs79129003	rs78912308	chr3:193373936	0.94	Cells - Cultured fibroblasts	A=0.096	-0.2407	2.90E-22	G	A	1621580	0.90	-0.00444	5.30E-03
rs79129003	rs78912308	chr3:193373936	0.94	Whole Blood	A=0.096	-0.1849	4.79E-07	G	A	1621580	0.90	-0.00444	5.30E-03
rs6444735	rs2935303	chr3:193374384	0.73	Whole Blood	T=0.47	-0.1046	3.15E-08	C	T	1701420	0.53	-0.00345	2.10E-04
rs6444735	rs4597663	chr3:193374384	0.96	Whole Blood	T=0.47	-0.1046	3.15E-08	C	T	1701420	0.53	-0.00345	2.10E-04
rs6444735	rs4597663	chr3:193374384	0.96	Cells - Cultured fibroblasts	T=0.47	-0.0614	3.21E-05	C	T	1701420	0.53	-0.00345	2.10E-04
rs6444735	rs2935303	chr3:193374384	0.73	Cells - Cultured fibroblasts	T=0.47	-0.0614	3.21E-05	C	T	1701420	0.53	-0.00345	2.10E-04
rs9851685	rs2935303	chr3:193374964	0.71	Cells - Cultured fibroblasts	C=0.475	-0.0610	3.76E-05	T	C	1800310	0.53	-0.00273	2.64E-03
rs9851685	rs4597663	chr3:193374964	0.98	Whole Blood	C=0.475	-0.1046	3.15E-08	T	C	1800310	0.53	-0.00273	2.64E-03
rs9851685	rs4597663	chr3:193374964	0.98	Cells - Cultured fibroblasts	C=0.475	-0.0610	3.76E-05	T	C	1800310	0.53	-0.00273	2.64E-03
rs9851685	rs2935303	chr3:193374964	0.71	Whole Blood	C=0.475	-0.1046	3.15E-08	T	C	1800310	0.53	-0.00273	2.64E-03
rs9837255	rs4597663	chr3:193376227	0.85	Cells - Cultured fibroblasts	T=0.439	-0.0669	4.39E-06	C	T	1645020	0.56	-0.00281	2.78E-03
rs9837255	rs4597663	chr3:193376227	0.85	Whole Blood	T=0.439	-0.0964	3.67E-07	C	T	1645020	0.56	-0.00281	2.78E-03
rs9837682	rs4597663	chr3:193376298	0.85	Whole Blood	A=0.439	-0.0964	3.67E-07	G	A	1800310	0.56	-0.0027	3.06E-03
rs9837682	rs4597663	chr3:193376298	0.85	Cells - Cultured fibroblasts	A=0.439	-0.0669	4.39E-06	G	A	1800310	0.56	-0.0027	3.06E-03
rs28457829	rs4597663	chr3:193377037	0.85	Cells - Cultured fibroblasts	A=0.439	-0.0669	4.39E-06	G	A	1701420	0.56	-0.00333	3.73E-04
rs28457829	rs4597663	chr3:193377037	0.85	Whole Blood	A=0.439	-0.0964	3.67E-07	G	A	1701420	0.56	-0.00333	3.73E-04
rs9291060	rs4597663	chr3:193378126	0.85	Cells - Cultured fibroblasts	C=0.439	-0.0669	4.39E-06	A	C	1701420	0.56	-0.00332	3.88E-04
rs9291060	rs4597663	chr3:193378126	0.85	Whole Blood	C=0.439	-0.0964	3.67E-07	A	C	1701420	0.56	-0.00332	3.88E-04
rs9813526	rs4597663	chr3:193381915	0.85	Whole Blood	C=0.439	-0.0957	3.90E-07	T	C	1800310	0.56	-0.00281	2.04E-03
rs9813526	rs4597663	chr3:193381915	0.85	Cells - Cultured fibroblasts	C=0.439	-0.0669	4.39E-06	T	C	1800310	0.56	-0.00281	2.04E-03
rs9831353	rs4597663	chr3:193382138	0.85	Whole Blood	G=0.439	-0.1000	8.78E-08	A	G	1800310	0.56	-0.00269	3.18E-03
rs9831353	rs4597663	chr3:193382138	0.85	Cells - Cultured fibroblasts	G=0.439	-0.0670	3.23E-06	A	G	1800310	0.56	-0.00269	3.18E-03
rs9869814	rs2935303	chr3:193382370	0.73	Whole Blood	A=0.47	-0.1039	3.38E-08	G	A	1800310	0.52	-0.00279	2.07E-03
rs9869814	rs4597663	chr3:193382370	0.96	Cells - Cultured fibroblasts	A=0.47	-0.0614	3.21E-05	G	A	1800310	0.52	-0.00279	2.07E-03
rs9869814	rs2935303	chr3:193382370	0.73	Cells - Cultured fibroblasts	A=0.47	-0.0614	3.21E-05	G	A	1800310	0.52	-0.00279	2.07E-03
rs9869814	rs4597663	chr3:193382370	0.96	Whole Blood	A=0.47	-0.1039	3.38E-08	G	A	1800310	0.52	-0.00279	2.07E-03
rs10937594	rs4597663	chr3:193383943	0.85	Whole Blood	C=0.439	-0.1007	8.13E-08	T	C	1800310	0.56	-0.00256	4.92E-03
rs10937594	rs4597663	chr3:193383943	0.85	Cells - Cultured fibroblasts	C=0.439	-0.0670	3.23E-06	T	C	1800310	0.56	-0.00256	4.92E-03
rs10804957	rs2935303	chr3:193383973	0.73	Whole Blood	G=0.47	-0.1046	3.15E-08	A	G	1701420	0.53	-0.00341	2.48E-04
rs10804957	rs2935303	chr3:193383973	0.73	Cells - Cultured fibroblasts	G=0.47	-0.0614	3.21E-05	A	G	1701420	0.53	-0.00341	2.48E-04

MTAG SNP	eQTL SNP	Position GRCH37	R^2	Tissue	Effect AF	Effect_Size	P_{eQTL}	a1	a2	N_{MTAG}	a1 frequency	Beta	P_{MTAG}
rs10804957	rs4597663	chr3:193383973	0.96	Cells - Cultured fibroblasts	G=0.47	-0.0614	3.21E-05	A	G	1701420	0.53	-0.00341	2.48E-04
rs10804957	rs4597663	chr3:193383973	0.96	Whole Blood	G=0.47	-0.1046	3.15E-08	A	G	1701420	0.53	-0.00341	2.48E-04
rs13070791	rs4597663	chr3:193385275	0.83	Whole Blood	T=0.434	-0.0969	3.30E-07	C	T	1800310	0.57	-0.00284	1.85E-03
rs13070791	rs4597663	chr3:193385275	0.83	Cells - Cultured fibroblasts	T=0.434	-0.0667	4.45E-06	C	T	1800310	0.57	-0.00284	1.85E-03
rs12494482	rs4597663	chr3:193386239	0.96	Cells - Cultured fibroblasts	T=0.47	-0.0614	3.21E-05	C	T	1800310	0.53	-0.00276	2.37E-03
rs12494482	rs4597663	chr3:193386239	0.96	Whole Blood	T=0.47	-0.1046	3.15E-08	C	T	1800310	0.53	-0.00276	2.37E-03
rs12494482	rs2935303	chr3:193386239	0.73	Whole Blood	T=0.47	-0.1046	3.15E-08	C	T	1800310	0.53	-0.00276	2.37E-03
rs12494482	rs2935303	chr3:193386239	0.73	Cells - Cultured fibroblasts	T=0.47	-0.0614	3.21E-05	C	T	1800310	0.53	-0.00276	2.37E-03
rs7616448	rs4597663	chr3:193387345	0.85	Whole Blood	A=0.439	-0.0964	3.67E-07	G	A	1800310	0.56	-0.00265	3.65E-03
rs7616448	rs4597663	chr3:193387345	0.85	Cells - Cultured fibroblasts	A=0.439	-0.0669	4.39E-06	G	A	1800310	0.56	-0.00265	3.65E-03
rs11915238	rs4597663	chr3:193387831	0.96	Whole Blood	C=0.47	-0.1038	4.35E-08	T	C	1800310	0.53	-0.00281	1.94E-03
rs11915238	rs4597663	chr3:193387831	0.96	Cells - Cultured fibroblasts	C=0.47	-0.0610	3.43E-05	T	C	1800310	0.53	-0.00281	1.94E-03
rs11915238	rs2935303	chr3:193387831	0.73	Cells - Cultured fibroblasts	C=0.47	-0.0610	3.43E-05	T	C	1800310	0.53	-0.00281	1.94E-03
rs11915238	rs2935303	chr3:193387831	0.73	Whole Blood	C=0.47	-0.1038	4.35E-08	T	C	1800310	0.53	-0.00281	1.94E-03
rs10937596	rs4597663	chr3:193387937	0.96	Whole Blood	A=0.47	-0.1046	3.15E-08	C	A	1800310	0.53	-0.00275	2.46E-03
rs10937596	rs4597663	chr3:193387937	0.96	Cells - Cultured fibroblasts	A=0.47	-0.0614	3.21E-05	C	A	1800310	0.53	-0.00275	2.46E-03
rs10937596	rs2935303	chr3:193387937	0.73	Whole Blood	A=0.47	-0.1046	3.15E-08	C	A	1800310	0.53	-0.00275	2.46E-03
rs10937596	rs2935303	chr3:193387937	0.73	Cells - Cultured fibroblasts	A=0.47	-0.0614	3.21E-05	C	A	1800310	0.53	-0.00275	2.46E-03
rs1985005	rs2935303	chr3:193388237	0.73	Whole Blood	T=0.47	-0.1041	3.46E-08	C	T	1800310	0.53	-0.00268	3.17E-03
rs1985005	rs2935303	chr3:193388237	0.73	Cells - Cultured fibroblasts	T=0.47	-0.0609	3.41E-05	C	T	1800310	0.53	-0.00268	3.17E-03
rs1985005	rs4597663	chr3:193388237	0.96	Cells - Cultured fibroblasts	T=0.47	-0.0609	3.41E-05	C	T	1800310	0.53	-0.00268	3.17E-03
rs1985005	rs4597663	chr3:193388237	0.96	Whole Blood	T=0.47	-0.1041	3.46E-08	C	T	1800310	0.53	-0.00268	3.17E-03
rs11720294	rs4597663	chr3:193389390	0.96	Cells - Cultured fibroblasts	A=0.47	-0.0609	3.41E-05	G	A	1800310	0.53	-0.00269	3.02E-03
rs11720294	rs4597663	chr3:193389390	0.96	Whole Blood	A=0.47	-0.1041	3.46E-08	G	A	1800310	0.53	-0.00269	3.02E-03
rs11720294	rs2935303	chr3:193389390	0.73	Cells - Cultured fibroblasts	A=0.47	-0.0609	3.41E-05	G	A	1800310	0.53	-0.00269	3.02E-03
rs11720294	rs2935303	chr3:193389390	0.73	Whole Blood	A=0.47	-0.1041	3.46E-08	G	A	1800310	0.53	-0.00269	3.02E-03
rs11720234	rs4597663	chr3:193389413	0.85	Whole Blood	A=0.439	-0.0975	2.91E-07	C	A	1701420	0.56	-0.00331	4.01E-04
rs11720234	rs4597663	chr3:193389413	0.85	Cells - Cultured fibroblasts	A=0.439	-0.0683	2.65E-06	C	A	1701420	0.56	-0.00331	4.01E-04
rs11720340	rs2935303	chr3:193389485	0.73	Whole Blood	A=0.47	-0.1030	4.94E-08	G	A	1645020	0.53	-0.00275	3.28E-03
rs11720340	rs4597663	chr3:193389485	0.96	Cells - Cultured fibroblasts	A=0.47	-0.0594	5.25E-05	G	A	1645020	0.53	-0.00275	3.28E-03

MTAG SNP	eQTL SNP	Position GRCH37	R^2	Tissue	Effect AF	Effect_Size	P_{eQTL}	a1	a2	N_{MTAG}	a1 frequency	Beta	P_{MTAG}
rs11720340	rs2935303	chr3:193389485	0.73	Cells - Cultured fibroblasts	A=0.47	-0.0594	5.25E-05	G	A	1645020	0.53	-0.00275	3.28E-03
rs11720340	rs4597663	chr3:193389485	0.96	Whole Blood	A=0.47	-0.1030	4.94E-08	G	A	1645020	0.53	-0.00275	3.28E-03
rs6796713	rs2935303	chr3:193391085	0.73	Whole Blood	A=0.47	-0.1019	6.62E-08	G	A	1800310	0.52	-0.0028	2.00E-03
rs6796713	rs4597663	chr3:193391085	0.96	Cells - Cultured fibroblasts	A=0.47	-0.0594	5.25E-05	G	A	1800310	0.52	-0.0028	2.00E-03
rs6796713	rs4597663	chr3:193391085	0.96	Whole Blood	A=0.47	-0.1019	6.62E-08	G	A	1800310	0.52	-0.0028	2.00E-03
rs6796713	rs2935303	chr3:193391085	0.73	Cells - Cultured fibroblasts	A=0.47	-0.0594	5.25E-05	G	A	1800310	0.52	-0.0028	2.00E-03
rs6444737	rs4597663	chr3:193391680	0.96	Whole Blood	G=0.47	-0.1034	4.24E-08	A	G	1800310	0.52	-0.00285	1.67E-03
rs6444737	rs2935303	chr3:193391680	0.73	Cells - Cultured fibroblasts	G=0.47	-0.0618	2.69E-05	A	G	1800310	0.52	-0.00285	1.67E-03
rs6444737	rs2935303	chr3:193391680	0.73	Whole Blood	G=0.47	-0.1034	4.24E-08	A	G	1800310	0.52	-0.00285	1.67E-03
rs6444737	rs4597663	chr3:193391680	0.96	Cells - Cultured fibroblasts	G=0.47	-0.0618	2.69E-05	A	G	1800310	0.52	-0.00285	1.67E-03
rs78898827	rs78912308	chr3:193392197	0.94	Whole Blood	G=0.096	-0.1830	9.05E-07	A	G	1621580	0.90	-0.0047	3.11E-03
rs78898827	rs78912308	chr3:193392197	0.94	Cells - Cultured fibroblasts	G=0.096	-0.2416	5.34E-22	A	G	1621580	0.90	-0.0047	3.11E-03
rs3851984	rs2935303	chr3:193393612	0.73	Cells - Cultured fibroblasts	G=0.47	-0.0614	3.21E-05	A	G	1800310	0.53	-0.00274	2.48E-03
rs3851984	rs4597663	chr3:193393612	0.96	Whole Blood	G=0.47	-0.1046	3.15E-08	A	G	1800310	0.53	-0.00274	2.48E-03
rs3851984	rs4597663	chr3:193393612	0.96	Cells - Cultured fibroblasts	G=0.47	-0.0614	3.21E-05	A	G	1800310	0.53	-0.00274	2.48E-03
rs3851984	rs2935303	chr3:193393612	0.73	Whole Blood	G=0.47	-0.1046	3.15E-08	A	G	1800310	0.53	-0.00274	2.48E-03
rs4443116	rs4597663	chr3:193401021	0.96	Whole Blood	G=0.47	-0.0983	1.75E-07	A	G	1800310	0.52	-0.00284	1.75E-03
rs4443116	rs2935303	chr3:193401021	0.73	Whole Blood	G=0.47	-0.0983	1.75E-07	A	G	1800310	0.52	-0.00284	1.75E-03
rs1806741	rs4597663	chr3:193401874	0.85	Whole Blood	T=0.439	-0.0958	4.02E-07	C	T	1800310	0.56	-0.00261	4.28E-03
rs1806741	rs4597663	chr3:193401874	0.85	Cells - Cultured fibroblasts	T=0.439	-0.0655	6.83E-06	C	T	1800310	0.56	-0.00261	4.28E-03
rs2367721	rs4597663	chr3:193402003	0.85	Cells - Cultured fibroblasts	G=0.439	-0.0635	1.30E-05	A	G	1800310	0.56	-0.00267	3.40E-03
rs2367721	rs4597663	chr3:193402003	0.85	Whole Blood	G=0.439	-0.0948	5.36E-07	A	G	1800310	0.56	-0.00267	3.40E-03
rs885053	rs4597663	chr3:193402008	0.85	Whole Blood	C=0.439	-0.1007	8.13E-08	T	C	1701420	0.56	-0.00316	7.30E-04
rs885053	rs4597663	chr3:193402008	0.85	Cells - Cultured fibroblasts	C=0.439	-0.0670	3.23E-06	T	C	1701420	0.56	-0.00316	7.30E-04
rs6444739	rs4597663	chr3:193403048	0.85	Cells - Cultured fibroblasts	A=0.439	-0.0669	4.39E-06	G	A	1800310	0.56	-0.00283	1.93E-03
rs6444739	rs4597663	chr3:193403048	0.85	Whole Blood	A=0.439	-0.0952	4.86E-07	G	A	1800310	0.56	-0.00283	1.93E-03
rs79911093	rs78912308	chr3:193405301	0.94	Whole Blood	A=0.096	-0.1849	4.79E-07	G	A	1621580	0.90	-0.00461	3.77E-03
rs79911093	rs78912308	chr3:193405301	0.94	Cells - Cultured fibroblasts	A=0.096	-0.2407	2.90E-22	G	A	1621580	0.90	-0.00461	3.77E-03
rs79911093	rs78912308	chr3:193405301	0.94	Pancreas	A=0.096	-0.3050	3.79E-05	G	A	1621580	0.90	-0.00461	3.77E-03
rs12696674	rs4597663	chr3:193407447	0.85	Cells - Cultured fibroblasts	T=0.439	-0.0669	4.39E-06	C	T	1800310	0.56	-0.00281	2.03E-03

MTAG SNP	eQTL SNP	Position GRCH37	R^2	Tissue	Effect AF	Effect_Size	P_{eQTL}	a1	a2	N_{MTAG}	a1 frequency	Beta	P_{MTAG}
rs12696674	rs4597663	chr3:193407447	0.85	Whole Blood	T=0.439	-0.0952	4.86E-07	C	T	1800310	0.56	-0.00281	2.03E-03
rs13068219	rs4597663	chr3:193407650	0.85	Whole Blood	A=0.439	-0.0958	4.02E-07	G	A	1800310	0.56	-0.00263	3.96E-03
rs13068219	rs4597663	chr3:193407650	0.85	Cells - Cultured fibroblasts	A=0.439	-0.0655	6.83E-06	G	A	1800310	0.56	-0.00263	3.96E-03
rs13067959	rs4597663	chr3:193407703	0.85	Whole Blood	A=0.439	-0.0955	4.58E-07	C	A	1800310	0.56	-0.00286	1.72E-03
rs13067959	rs4597663	chr3:193407703	0.85	Cells - Cultured fibroblasts	A=0.439	-0.0673	4.14E-06	C	A	1800310	0.56	-0.00286	1.72E-03
rs6793945	rs4597663	chr3:193407889	0.85	Whole Blood	G=0.439	-0.0995	1.19E-07	A	G	1800310	0.55	-0.00271	2.92E-03
rs6793945	rs4597663	chr3:193407889	0.85	Cells - Cultured fibroblasts	G=0.439	-0.0672	3.36E-06	A	G	1800310	0.55	-0.00271	2.92E-03
rs7638928	rs4597663	chr3:193408419	0.85	Whole Blood	G=0.439	-0.0952	4.86E-07	A	G	1800310	0.56	-0.00282	1.95E-03
rs7638928	rs4597663	chr3:193408419	0.85	Cells - Cultured fibroblasts	G=0.439	-0.0669	4.39E-06	A	G	1800310	0.56	-0.00282	1.95E-03
rs111999715	rs78912308	chr3:193411322	0.89	Pancreas	T=0.091	-0.3368	1.14E-05	G	T	1445630	0.90	-0.00552	7.71E-04
rs111999715	rs78912308	chr3:193411322	0.89	Whole Blood	T=0.091	-0.1853	7.34E-07	G	T	1445630	0.90	-0.00552	7.71E-04
rs111999715	rs78912308	chr3:193411322	0.89	Cells - Cultured fibroblasts	T=0.091	-0.2406	2.08E-21	G	T	1445630	0.90	-0.00552	7.71E-04
rs1056390	rs4597663	chr3:193414481	0.85	Whole Blood	T=0.439	-0.0956	3.86E-07	C	T	1701420	0.55	-0.00322	5.58E-04
rs1056390	rs4597663	chr3:193414481	0.85	Cells - Cultured fibroblasts	T=0.439	-0.0634	1.23E-05	C	T	1701420	0.55	-0.00322	5.58E-04
rs11719309	rs4597663	chr3:193414780	0.85	Whole Blood	C=0.439	-0.0956	3.86E-07	T	C	1645020	0.55	-0.00277	3.16E-03
rs11719309	rs4597663	chr3:193414780	0.85	Cells - Cultured fibroblasts	C=0.439	-0.0634	1.23E-05	T	C	1645020	0.55	-0.00277	3.16E-03
rs56329083	rs78912308	chr3:193415416	0.89	Whole Blood	G=0.091	-0.1873	3.85E-07	A	G	1621580	0.90	-0.00459	3.86E-03
rs56329083	rs78912308	chr3:193415416	0.89	Pancreas	G=0.091	-0.3404	6.44E-06	A	G	1621580	0.90	-0.00459	3.86E-03
rs56329083	rs78912308	chr3:193415416	0.89	Cells - Cultured fibroblasts	G=0.091	-0.2401	1.02E-21	A	G	1621580	0.90	-0.00459	3.86E-03
rs12152411	rs4597663	chr3:193415679	0.85	Whole Blood	C=0.439	-0.0956	3.86E-07	T	C	1800310	0.55	-0.00265	3.55E-03
rs12152411	rs4597663	chr3:193415679	0.85	Cells - Cultured fibroblasts	C=0.439	-0.0634	1.23E-05	T	C	1800310	0.55	-0.00265	3.55E-03
rs77918412	rs78912308	chr3:193416448	0.89	Pancreas	T=0.091	-0.3404	6.44E-06	C	T	1621580	0.90	-0.00465	3.39E-03
rs77918412	rs78912308	chr3:193416448	0.89	Cells - Cultured fibroblasts	T=0.091	-0.2401	1.02E-21	C	T	1621580	0.90	-0.00465	3.39E-03
rs77918412	rs78912308	chr3:193416448	0.89	Whole Blood	T=0.091	-0.1873	3.85E-07	C	T	1621580	0.90	-0.00465	3.39E-03
rs7652334	rs4597663	chr3:193420857	0.85	Whole Blood	C=0.439	-0.0896	2.50E-06	T	C	1800310	0.55	-0.00269	3.16E-03
rs7652334	rs4597663	chr3:193420857	0.85	Cells - Cultured fibroblasts	C=0.439	-0.0605	4.22E-05	T	C	1800310	0.55	-0.00269	3.16E-03
rs6787463	rs2935303	chr3:193318291	0.66	Whole Blood	A=0.49	-0.1020	6.22E-08	G	A	1800310	0.52	-0.00275	2.37E-03
rs6787463	rs4597663	chr3:193318291	0.92	Cells - Cultured fibroblasts	A=0.49	-0.0569	1.06E-04	G	A	1800310	0.52	-0.00275	2.37E-03
rs6787463	rs2935303	chr3:193318291	0.66	Cells - Cultured fibroblasts	A=0.49	-0.0569	1.06E-04	G	A	1800310	0.52	-0.00275	2.37E-03
rs6787463	rs4597663	chr3:193318291	0.92	Whole Blood	A=0.49	-0.1020	6.22E-08	G	A	1800310	0.52	-0.00275	2.37E-03

MTAG SNP	eQTL SNP	Position GRCH37	R^2	Tissue	Effect AF	Effect_Size	P_{eQTL}	a1	a2	N_{MTAG}	a1 frequency	Beta	P_{MTAG}
rs6764269	rs6764269	chr3:193422395	1.00	Whole Blood	C=0.591	-0.0857	4.52E-06	T	C	1800310	0.46	-0.00265	3.48E-03

Table S5. Summary of key published human evidence for OPA1 expression in MASLD and liver cirrhosis.

Source	Human tissue/cohort	Assay	Key OPA1 finding
Human Protein Atlas[6, 7]	Normal human tissues	RNA-seq / IHC	Moderate hepatic OPA1 protein staining and relatively high OPA1 RNA expression.
Da Dalt et al., 2023[8]	GSE135251 + GSE207310	Bulk RNA-seq reanalysis	Higher hepatic OPA1 mRNA; positive association with NAFLD Activity Score in both cohorts.
Moore et al., 2022[9]	n=130; control/NAFLD/borderline NASH/definite NASH	Western blot	Hepatic OPA1 protein decreased from NAFLD onset through NASH progression.
Niu et al., 2022[10]	Control n=15; NASH n=20; cirrhosis n=10	DIA-MS proteomics	No significant total OPA1 difference in NASH or cirrhosis versus control after adjusted analysis.
Zhao et al., 2013[11]	HCC/cirrhotic/non-cirrhotic liver tissues	Western blot	Indirect suggestion of lower OPA1 by the cirrhotic stage; no direct cirrhosis-versus-normal estimate.

Supplementary References

1. Sun, Z., et al., *Genetic variants in HFE are associated with non-alcoholic fatty liver disease in lean individuals.* JHEP Rep, 2023. **5**(7): p. 100744.
2. Ghodsian, N., et al., *Electronic health record-based genome-wide meta-analysis provides insights on the genetic architecture of non-alcoholic fatty liver disease.* Cell Rep Med, 2021. **2**(11): p. 100437.
3. Fairfield, C.J., et al., *Genome-Wide Association Study of NAFLD Using Electronic Health Records.* Hepatol Commun, 2022. **6**(2): p. 297-308.
4. Kurki, M.I., et al., *FinnGen provides genetic insights from a well-phenotyped isolated population.* Nature, 2023. **613**(7944): p. 508-518.
5. Ghouse, J., et al., *Integrative common and rare variant analyses provide insights into the genetic architecture of liver cirrhosis.* Nat Genet, 2024. **56**(5): p. 827-837.
6. Uhlen, M., et al., *Proteomics. Tissue-based map of the human proteome.* Science, 2015. **347**(6220): p. 1260419.
7. *The Human Protein Atlas*. 2025-11-11 [cited 2026 2026-01-18]; Version 25.0:[Available from: https://www.proteinatlas.org/.
8. Da Dalt, L., et al., *The inhibition of inner mitochondrial fusion in hepatocytes reduces non-alcoholic fatty liver and improves metabolic profile during obesity by modulating bile acid conjugation.* Cardiovasc Res, 2024. **119**(18): p. 2917-2929.
9. Moore, M.P., et al., *Compromised hepatic mitochondrial fatty acid oxidation and reduced markers of mitochondrial turnover in human NAFLD.* Hepatology, 2022. **76**(5): p. 1452-1465.
10. Niu, L., et al., *Dynamic human liver proteome atlas reveals functional insights into disease pathways.* Mol Syst Biol, 2022. **18**(5): p. e10947.
11. Zhao, X., et al., *OPA1 downregulation is involved in sorafenib-induced apoptosis in hepatocellular carcinoma.* Lab Invest, 2013. **93**(1): p. 8-19.
